# IRAK3 modulates downstream innate immune signalling through its guanylate cyclase activity

**DOI:** 10.1038/s41598-019-51913-3

**Published:** 2019-10-29

**Authors:** L. A. Freihat, J. I. Wheeler, A. Wong, I. Turek, D. T. Manallack, H. R. Irving

**Affiliations:** 1 Monash Institute of Pharmaceutical Sciences, Monash University, Parkville, VIC 3052 Australia; 2 La Trobe Institute for Molecular Science, La Trobe University, Bendigo, VIC 3552 Australia; 3 AgriBio, La Trobe University, Bundoora, VIC, 3083 Australia; 4 Division of Biological and Environmental Science and Engineering, King Abdullah University of Science and Technology, Thuwal, Kingdom of Saudi Arabia; 5 Department of Biology, Wenzhou-Kean University, 88 Daxue Road, Ouhai, Wenzhou, Zhejiang Province 325060 China

**Keywords:** Extracellular signalling molecules, NF-kappaB

## Abstract

Interleukin-1 receptor associated kinase 3 (IRAK3) is a cytoplasmic homeostatic mediator of inflammatory responses and is potentially useful as a prognostic marker in inflammation. IRAK3 inhibits signalling cascades downstream of myddosome complexes associated with toll like receptors. IRAK3 contains a death domain that interacts with other IRAK family members, a pseudokinase domain and a C-terminus domain involved with tumour necrosis factor receptor associated factor 6 (TRAF6). Previous bioinformatic studies revealed that IRAK3 contained a guanylate cyclase centre in its pseudokinase domain but its role in IRAK3 action is unresolved. We demonstrate that wildtype IRAK3 is capable of producing cGMP. Furthermore, we show that a specific point mutation in the guanylate cyclase centre reduced cGMP production. Cells containing toll like receptor 4 and a nuclear factor kappa-light-chain-enhancer of activated B cells (NFĸB) reporter system were transfected with IRAK3 or mutant IRAK3 proteins. Cell-permeable cGMP treatment of untransfected control cells suppresses downstream signalling through modulation of the NFĸB in the presence of lipopolysaccharides. Cells transfected with wildtype IRAK3 also suppress lipopolysaccharide induced NFĸB activity in the absence of exogenous cGMP. Lipopolysaccharide induced NFĸB activity was not suppressed in cells transfected with the IRAK3 mutant with reduced cGMP-generating capacity. Whereas in the presence of exogenously applied cell-permeable cGMP the IRAK3 mutant was able to retain its function by suppressing lipopolysaccharide induced NFĸB activity. Furthermore, increasing the amount of membrane permeable cGMP did not affect IRAK3’s ability to reduce NFĸB activity. These results suggest that cGMP generated by IRAK3 may be involved in regulatory function of the protein where the presence of cGMP may selectively affect downstream signalling pathway(s) by modulating binding and/or activity of nearby proteins that interact in the inflammatory signalling cascade.

## Introduction

Cyclic nucleotides are important ubiquitous signalling molecules mediating numerous cellular responses regulated by sophisticated cellular management of cyclic nucleotide pools. For instance, cyclic adenosine monophosphate (cAMP) is confined to specific subcellular compartments formed by cellular protein complexes where it modulates function of associated target molecules^1–3^. Similarly cGMP is also compartmentalised in cardiomyocytes^4^. Several receptor kinases in plants shown to have guanylate cyclase activity potentially develop restricted cGMP-rich pockets within the cell to form a signalling microenvironment^5,6^. The plant receptor kinases are unusual in that they contain a guanylate cyclase centre within their intracellular kinase domain^7,8^. Autophosphorylation of the kinase domain and cGMP, the product of the guanylate cyclase centre, both regulate the activity of the plant receptors^8–10^. Previously we reported that the mammalian protein interleukin 1 receptor associated kinase 3 (IRAK3) contained a guanylate cyclase centre in its pseudokinase domain and the recombinant protein generated cGMP^11^.

The IRAK family of cytoplasmic proteins mediates the downstream signalling pathways from the family of toll like receptor (TLR) and interleukin-1 receptor (IL-1R) in innate immunity. IRAK1, IRAK2 and IRAK4 work in concert to stimulate myeloid differentiation primary response 88 (MyD88) dependent activation of nuclear factor kappa-light-chain enhancer of activated B cells (NFκB) and thus pro-inflammatory cytokine production^12^. Activation of all TLRs, except TLR3, involves the adaptor protein MyD88 and the formation of a myddosome complex containing MyD88, IRAK4, IRAK1 and/or IRAK2 interacting by the death domains common to these proteins^13,14^. Tumour necrosis factor receptor associated factor 6 (TRAF6) interacts with IRAK1/2 causing dissociation of the myddosome complex from the TLR and downstream this eventually stimulates transforming growth factor beta kinase 1 (TAK1) dependent activation of NFκB^13,14^. IRAK3 (also known as IRAK-M as it is mainly found in monocytes) acts to suppress the TLR signalling resulting in decreased pro-inflammatory cytokine production^15^. IRAK3 interferes with TAK1 dependent activation of NFκB, principally by preventing the dissociation of the IRAK-TRAF6 complexes from the myddosome. An alternative possibility is that IRAK3 directly interacts with the MyD88-IRAK myddosome and IRAK3 forms a complex with mitogen activated protein kinase kinase kinase 3 (MEKK3) and TRAF6 that stimulates MEKK3 dependent NFκB activation promoting expression of anti-inflammatory molecules^16–18^. The ligand concentration activating the TLR affects the NFκB pathways. Low doses of ligand favouring IRAK3-MEKK3 dependent NFκB activation and an overall inhibitory inflammation response whereas higher does promote TAK1 dependent NFκB activation at first with consequent inflammatory molecule production that is later suppressed by IRAK3 interacting with the myddosome^19^. Moreover, IRAK3 expression is up-regulated in response to dexamethasone and thus forms part of the glucocorticoid mediated immunosuppressive pathways^20^.

The free radical NO decreases TLR and IL-1R receptor dependent signal transduction by inhibiting the interactions between IRAK1 and TRAF6 thereby suppressing downstream TAK1 dependent NFκB activation^21^. NO binds to the haem group in soluble guanylate cyclases to stimulate cGMP production^22^. Increased cellular levels of cGMP achieved using the cGMP specific phosphodiesterase inhibitor sildenafil or the membrane permeable 8-bromo-cGMP or various knock outs implicate cGMP in anti-inflammatory effects in several different systems^23–29^. Changes in NFκB activity in human peripheral mononuclear cells have been shown to depend on cGMP, could be mimicked by 8-bromo-cGMP, were correlated with changes in expression of IL-6 and increased at lower and decreased at higher concentration of NO donors^30^. Additionally, inducible NO synthase and cGMP mediates lipopolysaccharide stimulated macrophage migration^31^. It is important to note that the effect of cGMP as an anti-inflammatory modulator is subject to species variation in addition to cell types. For instance, in rats cGMP induces tumour necrosis factor alpha (TNFα) production in Kupffer cells which is opposite to that observed in mice^32^ likely reflecting divergences in evolutionary development and current phenotypes^33^. The molecular pharmacological mechanisms underpinning the role of cGMP in inflammation are still only partly understood^34–38^.

Whether the guanylate cyclase centre in IRAK3^11^ contributes to its function is not known. Here we confirm the production of cGMP by recombinant IRAK3 and show that HEK 293 T cells transfected with IRAK3 have higher levels of cGMP. We interrogated the function of the guanylate cyclase centre of IRAK3 using site directed mutagenesis. Mutation of residues predicted to be key to the guanylate cyclase function modified cGMP production and correspondingly altered the effects of IRAK3 on downstream NFκB activity.

## Results

### IRAK3 generates cGMP

**Figure 1:**
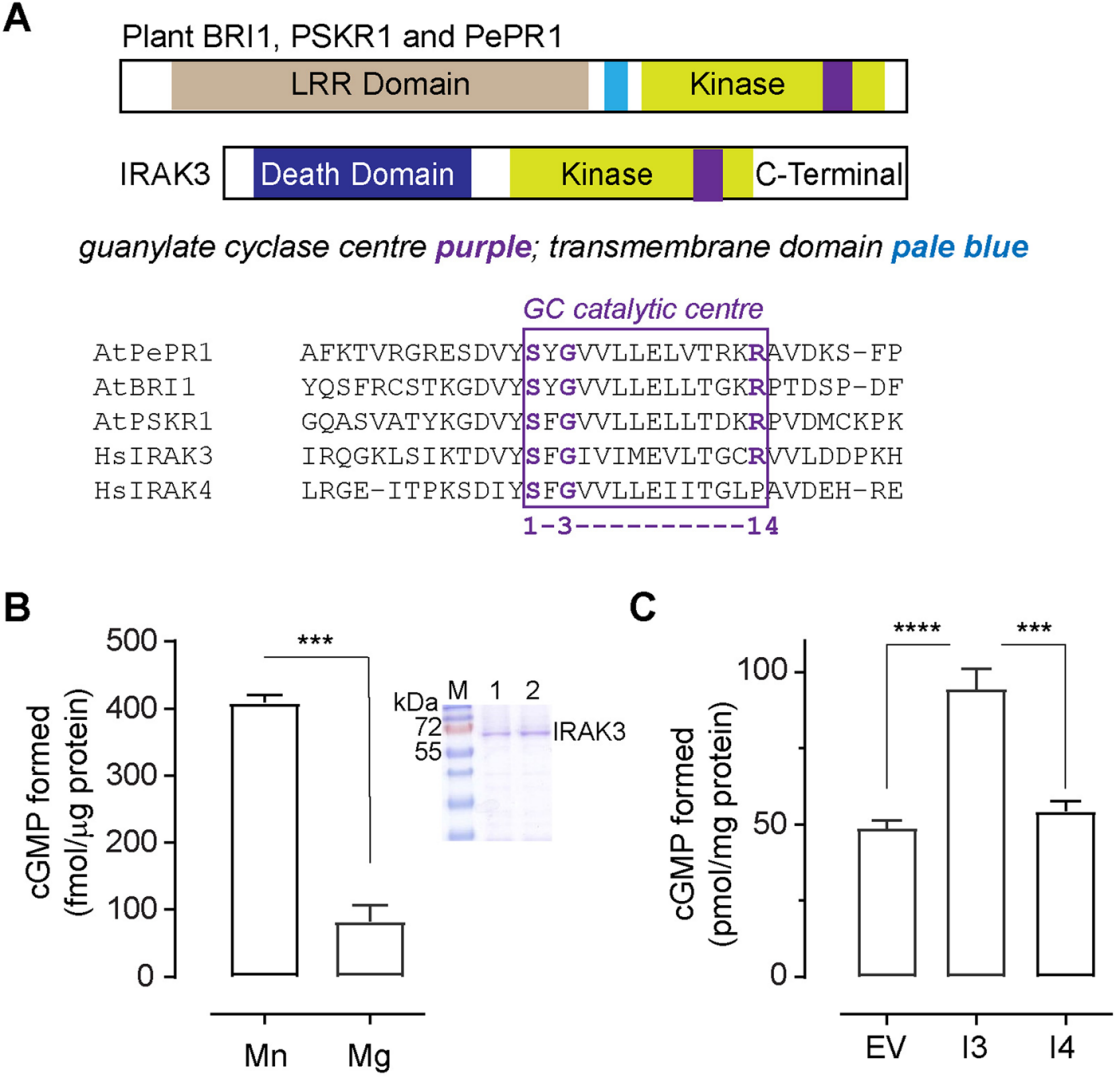
IRAK3 contains an active guanylate cyclase centre. (**A**) Domain architecture of human IRAK3 showing the guanylate cyclase centre (purple) and its alignment with known plant cryptic guanylate cyclases and IRAK4. The plant receptors contain an extracellular leucine rich repeat domain (brown), transmembrane domain (pale blue) and a kinase domain (yellow-green). IRAK3 is a cytoplasmic protein containing the signature death domain of the IRAK family, kinase (homology) domain and C-Terminal domain. Amino acids in purple are functionally assigned amino acids and IRAK4 lacks the essential basic amino acid at residue 14 of the catalytic centre previously described for plant guanylate cyclases^46,47^. Accession numbers for the aligned sequences are as follows: *Arabidopsis thaliana* (At) Perception of the Arabidopsis Danger Signal Peptide 1 (AtPePR1) Q9SSL9; *A*. *thaliana* brassinosteroid insensitive 1 (AtBRI1) O22476; *A*. *thaliana* phytosulfokine receptor 1 (AtPSKR1) Q9ZVR7; *Homo sapiens* interleukin 1 receptor associated kinase 3 (HsIRAK3) Q9Y616; HsIRAK4 Q9NWZ3. (**B**) Recombinant human IRAK3 generates cGMP and prefers Mn^2+^ ions over Mg^2+^ ions as a cofactor (mean ± sem, n = 6–8, ***P = 0.0001, unpaired t-test with Welch’s correction). The inset is of a Coomassie blue stained SDS-PAGE gel showing molecular weight markers (M) and purified recombinant IRAK3 protein at the expected molecular weight of ~68 kDa from two separate clones 1 and 2. (**C**) Manganese ions induce production of cGMP by IRAK3 in HEK 293 T cells. HEK 293 T cells were transiently transfected with vectors containing GFP only (EV), or IRAK3-GFP (I3) or IRAK4-GFP (I4) under the control of the cytomegalovirus (CMV) promoter. Cells were induced with 2 mM Mn^2+^ 24 hours post transfection for 10 min before the reaction was stopped and cGMP was measured (mean ± sem, n = 6–8, ****P < 0.0001 one-way ANOVA followed by Tukey’s multiple comparison test).

Pattern search motif analyses first revealed IRAK3 as containing a potential guanylate cyclase centre in its pseudokinase domain^11^. The amino acid sequence of this domain was aligned with sequences of the plant leucine rich repeat receptor kinase like proteins previously shown to contain an active guanylate cyclase centre. These include the kinase domains of the *Arabidopsis thaliana* perception of the Arabidopsis danger signal peptide 1 receptor (AtPePR1), *A*. *thaliana* brassinosteroid insensitive 1 (AtBRI1) and *A*. *thaliana* phytosulfokine receptor 1 (AtPSKR1)^7,8,39^ (Fig. 1A). Members of the IRAK family are cytosolic and each have a similar domain architecture where they all contain a death domain, a kinase homology domain and all, except IRAK4, contain a C-terminal domain. IRAK3 alone among the IRAK family members contains a guanylate cyclase centre (Fig. 1A). However, the other members of the IRAK family (IRAK1, IRAK3 and IRAK4) contain partial guanylate cyclase centres within the kinase homology domain (Supplementary Fig. 1A). An alignment comparing the amino acid sequence of IRAK3 close proximity to the guanylate cyclase centre shows that the sequence is conserved amongst other species (Supplementary Fig. 1B). The guanylate cyclase centre sequence found in IRAK3 is present in all the species. Particularly, within the mammalian species, the amino acid sequence is relatively conserved in the vicinity of the guanylate cyclase centre, which forms part of the pseudokinase domain.

Full length recombinant IRAK3 protein was prepared and its cofactor preference determined. Divalent cations such as Mg^2+^ and Mn^2+^ act as substrate cofactors or allosteric modulators for maximal activity of transmembrane and soluble guanylate cyclases, which can have different preferences^22^. The preferred cofactor for IRAK3 cGMP production is Mn^2+^ (Fig. 1B). Mn^2+^ was therefore used as the cofactor in subsequent experiments unless otherwise stated. A quantitative investigation using mass spectrometry was undertaken to confirm cGMP production by IRAK3 and compared to the immunoassays. Mass spectrometry cGMP analysis is more sensitive than the immunoassays^40–42^ and this can be seen by the approximately 10-fold increase in quantified cGMP content compared to the results obtained with the use of ELISA assay (Fig. 1B, Supplementary Fig. 2). The amount of cGMP generated *in vitro* by IRAK3 recombinant however is slightly less than the amount produced by the cytoplasmic domain of BRI1 (Supplementary Fig. 2)^10^ used as a reference protein to compare guanylate cyclase activity.

Since recombinant protein preparations of IRAK3 produce cGMP, it was of interest to see if such activity could be detected in cells. HEK 293 T cells do not express IRAK3^15,17,43^ therefore they were transiently transfected with vectors harbouring IRAK3-GFP or IRAK4-GFP genes and induced with Mn^2+^ 24 hours post transfection. In the presence of Mn^2+^, IRAK3 expressing cells generated nearly two-fold more cGMP compared to cells expressing GFP or IRAK4-GFP (Fig. 1C). Despite similarities in the amino acid sequence at the guanylate cyclase centre between IRAK3 and IRAK4, IRAK4 contains a proline at residue 14 rather than the typical positive arginine or lysine residue characteristic of a guanylate cyclase centre (Fig. 1A).

### Mutations in guanylate cyclase centre modify capability of IRAK3 to generate cGMP

**Figure 2:**
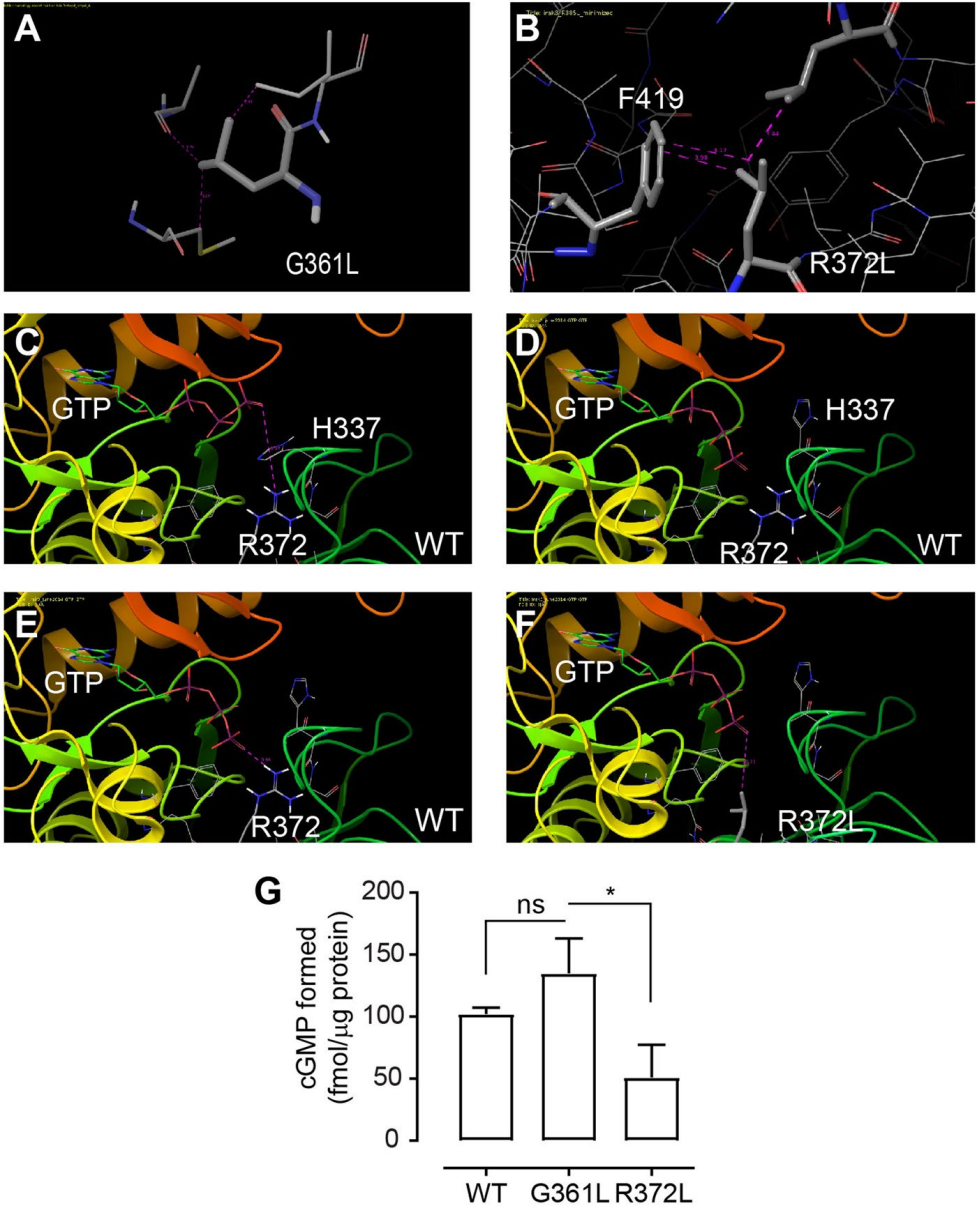
A mutation in the guanylate cyclase centre can suppress cGMP production by IRAK3. (**A**,**B**) Close up models of mutated versions of the human IRAK3 protein at the guanylate cyclase centre showing the G361L (**A**) and the R372L (**B**) mutations in the absence of GTP. (**C**–**E**) Close up of GTP docking interactions at the guanylate cyclase centre in wildtype (WT) human IRAK3. (**C**) Interaction between the γ phosphate of GTP and R372 in the wildtype protein when H337 interferes. (**D**) Interaction between the γ phosphate of GTP and R372 in the wildtype protein when H337 is moved. (**E**) Interaction between the γ phosphate of GTP and R372 in the wildtype protein at approximately 4 Å. (**F**) Close up of ribbon model of GTP docked into the guanylate cyclase centre of IRAK3 R372L mutant showing a lack of interaction with the L372 and the γ phosphate of GTP (residues separated by about 6 Å). (**G**) IRAK3 protein was expressed as either wildtype IRAK3, IRAK3 mutant G361L or IRAK3 mutant R372L. Mutant G361L produced comparable amounts of cGMP to IRAK3 wildtype. Mutant R372L showed significantly decreased cGMP production when compared to G361L mutant (mean ± sem, n = 3–5, *P = 0.0479, one-way ANOVA followed by Tukey’s multiple comparison test).

Mutations in the guanylate cyclase centre of PSKR1 and BRI1 have reduced the ability of the recombinant proteins to generate cGMP^8–10,44^. We initially took an *in silico* approach to identify if specific mutations altered the guanylate cyclase centre and vicinity using our homology model^11^ generated from the crystal structure of IRAK4^45^. We changed specific residues in the guanylate cyclase centre such as Glycine361 to Leucine (G361L) and Arginine372 to Leucine (R372L) and observed the effects on the overall structure and surrounding residues (Fig. 2A and B). The mutation of G361L representing residue number 3 in the guanylate cyclase centre of IRAK3 (Fig. 1A) resulted in a major steric clash, where the leucine side chain clashed with the adjacent C425 and A426. To relieve the clash the α-helix (413–425) may shift or alternatively a loop can commence from about residue 424 or earlier which may allow some space for the introduced leucine residue (Fig. 2A). The mutation of R372L representing residue 14 in the guanylate cyclase centre of IRAK3 (Fig. 1A), does not appear to have a major effect on the surrounding residues, as L372 may slip in adjacent to F419. The side chain of L372 can form a hydrophobic interaction with F419 and may still have an acceptable conformation so it may not have an effect on the overall structure (Fig. 2B). However, if the GTP molecule interacts with the arginine as predicted in the PSKR1 docking model^46^, then this may affect the affinity of GTP to the protein. A mutation at position 14 of the guanylate cyclase centre of PSKR1 effects binding to the phosphate acyl group and stabilises the transition of GTP to cGMP^46^. It is predicted that there may be an interaction between the GTP phosphate tail, which is negatively charged, and the positively charged arginine in the guanylate cyclase centre^47^. We docked GTP into our IRAK3 homology model and noted that the terminal phosphate groups of the GTP was about 9 Å away from the guanidine group of R372. In this position an interaction is unlikely, however this separation is due to H337 located in between the two groups of interest (Fig. 2C). To determine if an interaction between the phosphate group and R372 is possible, a number of manipulations were made. These included moving the H337 imidazole ring out of the way and adjusting the conformation of the triphosphate chain (Fig. 2D). This placed the phosphate and guanidine at a distance of close to 4 Å where an interaction may form between these residues, but this remains speculative and requires further experimental work (Fig. 2E). However, when R372 was mutated to leucine and a GTP dock was done on the R372L mutant, it is unlikely that the methyl group of the leucine can interact with the polar phosphate group of GTP (Fig. 2F). Therefore, from this docking result we predicted that mutant R372L may no longer be able to catalyse the production of cGMP due to the decreased chance of interaction and stabilisation of the GTP.

To test these predictions, we created IRAK3 G361L and IRAK3 R372L using site directed mutagenesis. Wildtype and mutant recombinant IRAK3 proteins were expressed in bacteria and cGMP production was measured. The G361L mutation had no major effect on cGMP production, since significant amounts of cGMP was produced comparable to wildtype IRAK3 (Fig. 2G). This finding indicates that the steric clash imposed by introduction of the G361L mutation in our homology-based model (Fig. 2A) is relieved and does not impact the guanylate cyclase activity of the mutant as the catalytic activity of the mutated protein is comparable to the activity of the wildtype IRAK3. The modelling study was able to reconcile this result as the leucine side chain could be readily accommodated within the protein structure (Fig. 2A). In addition, the IRAK R372L mutation did not generate significant levels of cGMP (Fig. 2G) which was in accord with the docking study which predicted a decrease in catalysis (Fig. 2F).

### cGMP is important in IRAK3 cellular function

HEK BLUE™ hTLR4 cells express the human toll-like receptor 4 (TLR4), lymphocyte antigen 96 (MD-2) and cluster of differentiation 14 (CD14) co-receptor genes, and an inducible secreted embryonic alkaline phosphatase (SEAP) reporter gene. The SEAP reporter gene is under the control of the IL-12 p40 promoter fused to five NFκB and AP-1 (activator protein 1) binding sites. Upon activation of TLR4 by lipopolysaccharide, induction of NFĸB occurs, which subsequently induces the SEAP reporter to produce alkaline phosphatase. The alkaline phosphatase released into the cell culture media causes quantifiable changes in colour of the QUANTI Blue reagent (Supplementary Fig. 3A). A dose response curve shows that significant increases in NFĸB activity occur in the presence of 0.1 ng/μl, with a maximal effect seen between 1–10 ng/μl lipopolysaccharide (Supplementary Fig. 3B). Transfection of HEK BLUE™ hTLR4 cells with wildtype IRAK3-GFP expressing plasmid, compared with transfection of the cells with GFP-expressing plasmid, suppressed NFĸB activity (Supplementary Fig. 3C,D) confirming prior work that IRAK3 acts as a negative regulator of NFĸB^15^ and demonstrates the viability of our experimental system.

**Figure 3:**
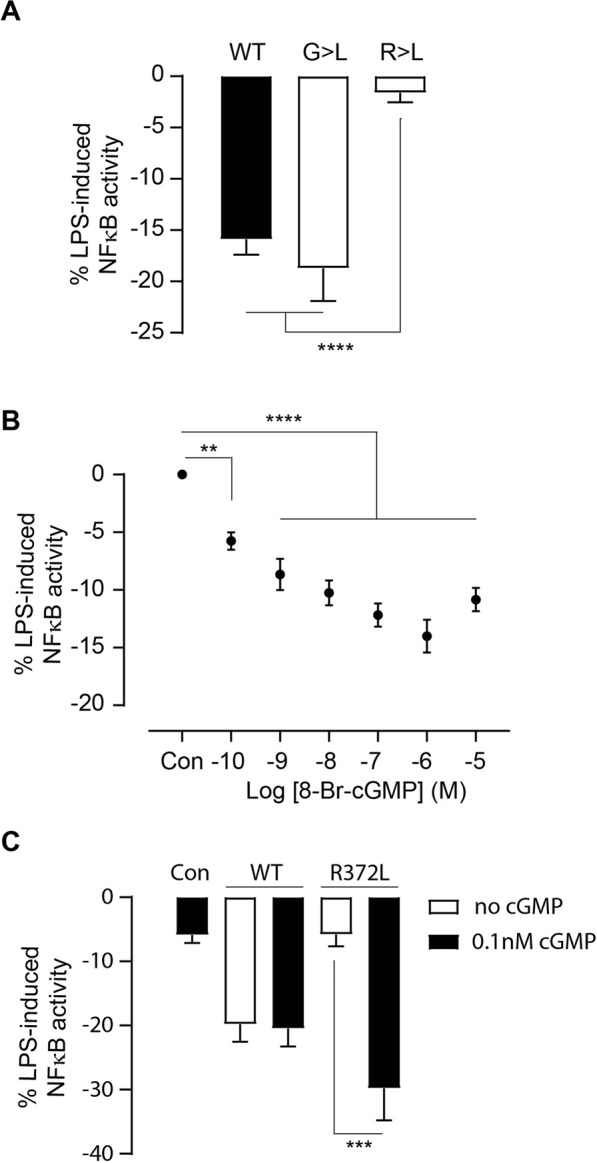
Effects of wild type and mutant IRAK3 proteins on NFκB activity. (**A**) HEK BLUE™ hTLR4 cells were transfected with plasmid expressing C-terminally GFP-tagged wildtype (WT) IRAK3, IRAK3 mutant G361L (G > L) or IRAK3 mutant R372L (R > L). Cells were induced with lipopolysaccharide (LPS, 10 ng/µl) and after 24 hours SEAP activity correlating to NFκB induction was measured and the activity was normalised to the control vector containing GFP only. Wildtype IRAK3 and IRAK3 mutant G361L both suppressed NFκB activity (mean ± sem, n = 15–30 from at least 3 separate experiments, ****P < 0.0001, one-way ANOVA followed by Tukey’s multiple comparison test). (**B**) Effects of different concentrations of 8-bromo-cGMP (8-Br-cGMP) on NFκB activity in HEK BLUE™ hTLR4 cells. Cells were induced with LPS (10 ng/µl) and after 24 hours SEAP activity corresponding to NFκB induction was measured and normalized to control cells (Con) not treated with 8-bromo-cGMP (mean ± sem, n = 4, **P < 0.01, ****P < 0.0001, one-way ANOVA followed by Dunnett’s multiple comparison test). (**C**) Low levels of 8-Br-cGMP (0.1 nM) alter the effect of IRAK3 mutant R372L on LPS-induced NFκB activity in transfected HEK BLUE™ hTLR4 cells. Cells were transfected with vector harbouring only GFP (Con) or C-terminally GFP-tagged wildtype (WT) IRAK3 or IRAK3 mutant R372L (R372L). Cells were induced with LPS (10 ng/µl) in the presence or absence of 0.1 nM 8-Br-cGMP and after 24 hours SEAP activity correlating to NFκB induction was measured and normalized to Con. Addition of 8-Br-cGMP made no difference to NFκB activity in cells transfected with WT IRAK3 (mean ± sem, n = 3, P = 0.8786, t-test with Welch’s correction). However, in cells transfected with IRAK3 mutant R372L (R372L), 8-Br-cGMP restored the inhibitory capacity of IRAK3 at levels which it alone did not have a marked effect as shown in Fig. 2B (mean ± sem, n = 11–12, ***P = 0.0008, t-test with Welch’s correction).

Modelling and recombinant protein studies (Fig. 2) indicate that mutations in the IRAK3 guanylate cyclase centre modified cGMP production. Therefore, to test if the ability of these mutants to form cGMP affected the function of IRAK3, IRAK3 wildtype and IRAK3 mutants G361L and R372L were expressed in HEK BLUE hTLR4 cells. Cells were checked for transfection efficiency using qualitative confocal microscopy and in parallel experiments, similar levels of IRAK3 mRNA was detected using quantitative PCR (Supplementary Fig. 4). Cells expressing the IRAK3 G361L mutant showed a reduction of NFĸB activity comparable to that seen with wildtype IRAK3 (Fig. 3A). The IRAK3 R372L mutant containing cells however, functioned as though IRAK3 was not present and did not reduce NFĸB activity (Fig. 3A), implicating this residue as potentially important for function of IRAK3 in modulating lipopolysaccharide induced NFĸB activity.

The IRAK3 R372L mutant has a suppressed ability to make cGMP (Fig. 2G) and also fails to decrease lipopolysaccharide induced NFĸB activity raising the possibility that cGMP may contribute to the normal suppression of NFĸB activity by IRAK3. Membrane-permeable cGMP (8-bromo-cGMP) had no effect on NFĸB activity in non-transfected HEK BLUE hTLR4 cells (Supplementary Fig. 5A). When these cells were induced with lipopolysaccharide in the presence of 8-bromo-cGMP, a significant suppression of NFĸB activity is observed (Fig. 3B). Since IRAK3 expressing HEK BLUE hTLR4 cells suppressed lipopolysaccharide-induced NFĸB activity (Fig. 3A), we explored whether excess 8-bromo-cGMP enhanced the effect of IRAK3 on the suppression of NFĸB activity. The IRAK3 inhibitory effect on NFĸB was not augmented by a sub-nanomolar concentration of cell permeable 8-bromo-cGMP (Fig. 3C). The IRAK3 mutant R372L does not reduce NFĸB activity stimulated by lipopolysaccharide unlike wildtype IRAK3 (Fig. 3A). To see if the negligible effect of the IRAK3 R372L mutant on suppression of NFKB activity can be reversed, we added sub-nanomolar amounts of cGMP during lipopolysaccharide induction. Significantly when 8-bromo-cGMP is present, the IRAK3 R372L mutant suppresses NFĸB activity just like the wildtype (Fig. 3C). A sub-nanomolar concentration of 8-bromo-cGMP was primarily selected to investigate the effect of added cGMP on IRAK3 and NFκB activity, as higher concentrations of cGMP may mask or overpower the effect of IRAK3 itself on NFĸB activity. A significant reduction in NFĸB activity in IRAK3 R372L expressing cells is observed from 0.0001 to 1 μM cGMP (Supplementary Fig. 5B).

## Discussion

IRAK3 is an important down regulator of inflammation that acts in part by decreasing transcription of cytokines induced by NFκB^15,18^. A conserved guanylate cyclase centre within the pseudokinase domain of IRAK3 allows IRAK3 to generate cGMP *in vitro*^11^. Mutations to the guanylate cyclase centre in IRAK3 modulate cGMP production *in vitro*. Cells transfected with plasmid expressing IRAK3 generate greater levels of cGMP than those transfected with plasmid expressing IRAK4, which lacks some of the key residues in the guanylate cyclase centre (Fig. 1). To test if cGMP contributed to the IRAK3 signal cascade, we used HEK BLUE NFκB reporter cells containing the human TLR4 responsive to bacterial lipopolysaccharide (Supplementary Fig. 3A). Lipopolysaccharide treatment stimulates NFκB activity and cells expressing IRAK3 suppress lipopolysaccharide dependent NFκB activation. However, the IRAK3 R372L mutant lacking the ability to generate cGMP only suppresses NFκB activity in the presence of cGMP (Fig. 3). Together these results highlight that IRAK3 acts to generate its nano-environment signalling molecule cGMP that in turn modulates IRAK3’s actions on the signal cascade inducing NFκB.

Once TLR/IL-1R are activated there is a sequence of molecular recruitments that occur, which involve the assembly of signalling molecules and complexes. This includes MyD88 a necessary adaptor protein in the TLR/IL-1R signalling cascade, subsequently recruiting the IRAK proteins (IRAK1, 2, 4). The MyD88-IRAK4-IRAK2 death domain myddosome complex interact via the death domain. Formation of these myddosomes allow the kinase domain of the IRAK proteins to be in close proximity for both trans and autophosphorylation and activation^14^. IRAK3 negatively regulates this pathway; it also binds via its death domain to the other members of the IRAK family, in other words, IRAK3 forms heterodimers with IRAK1 and IRAK2 to disable downstream NFκB activation and consequent inflammatory response^14^. IRAK3 expression is upregulated by the soluble guanylate cyclase stimulator NO and in human monocytes this suppresses lipopolysaccharide induced cytokine production^48,49^. The increases in IRAK3 levels contribute to the inhibitory effect of NO on the interaction between IRAK1 and TRAF6 and thus NFκB activation induced by lipopolysaccharide in macrophages^21^. However, IRAK3 contributes to only part of this process as membrane permeable cGMP suppresses lipopolysaccharide induced NFĸB activity in a dose-dependent fashion in untransfected HEK BLUE NFκB reporter cells not expressing IRAK3 (Fig. 3B) possibly via stabilising nuclear factor of kappa light polypeptide gene enhancer in B-cells inhibitor, alpha (IκBα)^50^. In the absence of lipopolysaccharide, NFĸB is not induced and membrane permeable cGMP has no effect confirming earlier reports^50,51^.

IRAK3 was initially identified in a pattern search for proteins containing guanylate cyclase centres^11^. The guanylate cyclase is embedded in the pseudokinase domain of IRAK3 and is conserved amongst the different IRAK3 molecules from fish to humans. Other members of the IRAK family have some similarities to guanylate cyclase centre but lack essential conserved amino acids in the search motif and the regions surrounding the guanylate cyclase centre that are predicted to be essential for catalysis^7,46,47,52^. For instance, both IRAK2 and IRAK4 contain a proline at position 14 of the guanylate cyclase centre (Supplementary Fig. 1) where a positively charged residue is required to orientate GTP into a binding pose suitable for catalysis^7,47,52^. Although the guanylate cyclase centre in IRAK1 contains key amino acids for catalysis at positions 1, 3 and 14 of the motif and has physicochemical properties that closely resemble that of currently known guanylate cyclases^53^, it however lacks an acidic residue, aspartic acid or glutamic acid, that is required for binding to cofactors such as Mg^2+^ or Mn^2+^ ions. This amino acid normally appears at two or three residues downstream of the catalytic centre which in the case of IRAK1, is occupied by a positively charged lysine residue^46^.

Interestingly, the IRAK3 guanylate cyclase centre resembles a group of novel non-canonical leucine rich repeat receptor-like kinases in plants containing guanylate cyclase centres embedded in the kinase domain including the well-characterized hormone receptors, PSKR1 and BRI1^6,54^. Mutations to key amino acids in the guanylate cyclase centre inhibited cGMP production by these proteins and also affected their cellular and biological functions^8,10,55^. For instance, the phosphorylation capability of BRI1 was found to be linked to the guanylate cyclase as the BRI^1815–1196^ KR1083/4AA mutant which yielded reduced guanylate cyclase activity, also showed reduced ability to phosphorylate the BR13 substrate^10^. In plants, these guanylate cyclase containing multidomain protein complexes also form dimers and this has added another layer of regulatory complexity in what seemed to be a high degree of cross-talk between the phosphorylation and cGMP generation^9,10,44^. Given the evidence from plants, it is therefore tempting to speculate that the marked conservation of guanylate cyclase centre in IRAK3 but not in other members of the IRAK family, presents significant biological implications since regions conserved due to low mutation rates are usually functionally important^56,57^. Consistently, a previous report has also hinted at a common biological role for such guanylate cyclases in mammals where alignment of IRAK3 orthologs revealed the presence of this guanylate cyclase centre in 19 out of 20 orthologous mammalian proteins^53^. As such, our results which linked IRAK3 guanylate cyclase activity to the NFĸB LPS-induced activity, has drawn interesting parallels in biological roles with plant systems where guanylate cyclases of such nature, also participates in immune response.

Recombinant IRAK3 generates cGMP and this appears to be dependent upon the guanylate cyclases centre as the mutation R372L has a reduced capacity to form cGMP. Although the amount of cGMP produced by IRAK3 is low (Fig. 1B,C), it is of similar order of magnitude to that generated by the recombinant kinase domains of PSKR1 and BRI1 (Supplementary Fig. 2)^10,11^. The reasons for low amounts of cGMP are not understood but can possibly be attributed to lack of essential cofactors and possibly interacting proteins in the *in vitro* assays. Another possibility is that IRAK3 and the other proteins may essentially only generate low levels of cGMP to generate a small change in the surrounding nano-environment of the protein^6^. The fact that cells expressing IRAK3 are induced to produce higher levels of cGMP than those transfected with the empty vector or IRAK4 (Fig. 1C) provides further evidence that IRAK3 can generate cGMP. There are similarities in the guanylate cyclase centre sequence between IRAK3 and IRAK4 but IRAK4 contains proline instead of arginine or lysine at the 14^th^ position of the guanylate cyclase centre (Fig. 1A) and is thus not predicted to have guanylate cyclase activity. A positively charged residue is important for docking the substrate GTP^46,47,53,58,59^. GTP cannot be docked into the IRAK3 R372L mutation and the actual mutant protein has reduced guanylate cyclase activity (Fig. 2G).

Increases in intracellular cGMP suppress the lipopolysaccharide induced TLR4 mediated signal cascade involving NFĸB^30^ that is also inhibited by IRAK3^15,43,60^. All the IRAK proteins contain a distinctive death domain involved in protein-protein interactions essential in forming the myddosome. IRAK1 and IRAK4 are active kinases that initiate a phosphorylation cascade, while IRAK2 is a pseudokinase and together they facilitate activation of NFκB to upregulate expression of pro-inflammatory cytokines^13,61^. We used the HEK BLUE hTLR4 NFĸB reporter system to study effects of wildtype and IRAK3 mutants on lipopolysaccharide induced NFĸB activity. Since HEK293 cells do not contain IRAK3^15,43^, this model avoids any confounding effects related to endogenous IRAK3 expression. Membrane permeable cGMP suppresses lipopolysaccharide induced NFĸB activity in a dose-dependent fashion in untransfected cells not expressing IRAK3 (Fig. 3B). Wildtype IRAK3 suppresses NFĸB activity as does the mutant G361L (Fig. 3A) that retains the capacity to produce cGMP (Fig. 2G) in the absence of any membrane permeable cGMP. It is noteworthy that IRAK3 R372L does not suppress NFĸB activity even though it is present but when it is supplemented with sub-nanomolar levels of membrane permeable cGMP, suppression of NFĸB activity is restored. The sub-nanomolar (0.1 nM) levels of cGMP alone had a small 5.8% suppressive effect on lipopolysaccharide induced NFĸB activity similar to that of the IRAK3 R372L mutant (Fig. 3C). However, when present together, there is an enhanced suppression of NFĸB activity to 29.8%, which exceeds that generated by wildtype IRAK3 alone or with sub-nanomolar cGMP (Fig. 3C). These results indicate that a synergistic interaction is occurring between the sub-nanomolar cGMP and the mutant IRAK3 protein and that this is required to restore the suppressive response of IRAK3 on NFĸB activity. IRAK3 interacts with the myddosome complex and inhibits the dissociation of IRAK1/IRAK2 thus preventing NFĸB activation^17,18^. The death domain is important in binding to proteins with death domains such as other members of the IRAK family and has been previously implicated in IRAK3 function. Alternate but specific surface residues in the death domain of IRAK3 are important in binding to IRAK2 or IRAK4^17^. Here using the IRAK3 R372L mutant, we obtained the first indication that the guanylate cyclase centre in the pseudokinase domain also contributes to IRAK3 function. The IRAK3 R372L mutant contains an intact death domain and therefore is unlikely to alter binding to IRAK2 or IRAK4.

Together these findings indicate that the ability of IRAK3 to generate cGMP may contribute to its function of suppressing NFĸB activity. Membrane permeable cGMP is necessary to enable IRAK3 372L mutant to reduce NFĸB activity but increasing these amounts from sub-nanomolar to micromolar did not further enhance this effect (Supplementary Fig. 5B). Adding sub-nanomolar amounts of membrane permeable cGMP did not enhance the ability of wildtype IRAK3 to suppress NFĸB activity (Fig. 3C). These results may be due to cGMP-independent regulatory mechanisms obscuring effects of cGMP in cells transfected with either wildtype or R372L mutant of IRAK3. Alternatively, our findings support the notion that only low amounts of cGMP are needed and that these may contribute to a nano-environment surrounding the IRAK3 myddosome complex enabling IRAK3 inhibition of IRAK1/2 disassociation. Such a localised scenario at the myddosome where IRAK3 generates cGMP is in keeping with the recently described cGMP nano-environments generated by natriuretic peptide receptors^4^. It is likely to represent a localised control system as distinct from a widespread control system managed by NO. NO can spread across the cell and activate soluble guanylate cyclases resulting in a wave of cGMP forming that inhibits NFĸB-induced cytokine production^21^.

## Materials and Methods

### Generation of IRAK3 and IRAK4 constructs

The IRAK3 gene in pBluescriptR (MHS1010-9204142, clone ID 30335802) was cloned into pDONR207 using Gateway® BP Clonase™ II Enzyme Mix (Invitrogen, Thermo Scientific USA). Five primers were designed to make four constructs; two forward primers, each containing the Kozak sequence and three reverse primers to either incorporate a Myc tag or a STOP codon (Supplementary Table 1) and these were generated by PCR. Heat shock transformation was used for all One Shot® Max Efficiency® DH5α™-T1R competent cells and bacteria were grown on LB plates or liquid broth containing the appropriate antibiotic (e.g. ampicillin 100 μg/ml or gentamycin 15 μg/ml). Plasmids were purified using PureLink® Quick Plasmid Miniprep Kit (Thermo Fisher Scientific, USA) for small scale preparations and PureLink® HiPure Plasmid Filter Midiprep Kit for larger scale preparations and DNA was quantitated with a Nanodrop® ND-1000 Spectrophotometer (Thermo Fischer Scientific, USA). Plasmids were sequenced (Micromon Monash University) and those with correct in frame insertions were kept and independently recombined into the appropriate Gateway expression vector, either pDEST17 for bacterial expression or pcDNA^TM^ 6.2/C-EmGFP-DEST or pcDNA-DEST40 for mammalian cell expression (Invitrogen, Thermo Scientific USA) using the Gateway® LR Clonase™ II Enzyme mix. Similarly, the IRAK4 gene in a pDONR223 plasmid (Plasmid 23749: PDONR223-IRAK4, Addgene)^62^ was transferred using the Gateway® LR Clonase™ II Enzyme Mix into the appropriate Gateway expression vector, either the pDEST17 for bacterial expression or pcDNA^TM^ 6.2/C-EmGFP-DEST or pcDNA-DEST40 for mammalian cell expression. For site directed mutagenesis, primers were designed using the Agilent Technologies Quick change primer design program to mutate specific residues in the kinase domain of the IRAK3 and IRAK4 gene (Supplementary Table 1). pDONR vectors containing IRAK3 or IRAK4 were used as templates and amplified with the specific primers (0.5 μM) by Phusion® High-Fidelity DNA Polymerase (initial denaturation at 98 °C for 30 seconds, followed by 20 cycles of 98 °C denaturation for 10 seconds, 68 °C annealing for 30 seconds and an extension at 70 °C for 4 min; final extension at 72 °C for 10 min). The PCR products were digested with the restriction enzyme DpnI (NEB, Australia) at 37 °C. Mutant clones were transformed into One Shot® Max Efficiency® DH5α™-T1R competent cells and grown in gentamicin (15 μg/ml) containing media for selection. Plasmids from specific clones were purified and confirmed by sequencing before being transferred in the appropriate expression vector via an LR reaction.

### Recombinant protein expression

The pDEST17 vector (Invitrogen) containing the gene of interest was used to express recombinant proteins (IRAK3, IRAK3G361L and IRAK3R372L) in BL21-AI *E*. *coli* cells (Invitrogen, USA) as previously described^63^. The BL21-AI *E*. *coli* system is designed for tight regulation and expression of toxic proteins from T7 promoter based systems under the control of the arabinose inducible *ara*BAD promoter^64–66^. High expressing cultures were selected and were upscaled to 500 ml liquid cultures grown to an OD600 of ~0.4 before induction with 0.2% L-arabinose (Sigma-Aldrich, Australia) and grown for a further 3 hours at 20 °C. Cells were harvested by centrifugation. Proteins were purified by affinity chromatography using the Ni-NTA agarose beads (QIAGEN, Germany) under native conditions following protocol 12 of the QIAexpressionist manual (QIAGEN, Germany) in the presence of 30 mM imidazole to decrease the unspecific binding onto the Ni-NTA beads and in the presence of complete EDTA-free protease inhibitor cocktail tablets (Roche, Australia). Eluted protein was concentrated using Vivaspin® 20 centrifugal concentrators (Sartorius Stedim Biotech, Germany) with a molecular weight cut-off of 30 kDa in the presence of 1 mM phenylmethylsulfonyl fluoride (PMSF) and protein was quantitated using a Nanodrop® ND-1000 Spectrophotometer protein at A_280_. Proteins were separated by SDS-PAGE using 16% separating and 4% stacking gels and run at 200 volts for 50 minutes (BioRAD mini electrophoresis setup), stained with Coomassie blue (0.03% (w/v) Coomassie Brilliant blue, 50% methanol, 10% glacial acetic acid and 40% distilled water), and destained with destain solution (40% methanol, 10% acetic acid and 50% distilled water).

### Cell Culture

Human embryonic kidney 293 T (HEK 293 T) cells (American Type Culture Collection ATCC: CRL-11268) and HEK BLUE™ hTLR4 cells (InvivoGen, USA) were grown in Dulbecco’s Modified Eagle Medium (DMEM; Gibco) with 10% (v/v) Foetal Bovine Serum (FBS; Gibco) in 5% CO_2_ at 37 °C in a tissue culture incubator. All cells were tested for mycoplasma before use; cells were split for 3 passages once taken from the frozen stock before testing for mycoplasma using the MycoAlert™ Mycoplasma Detection Kit (Lonza Australia). Cells were grown to 70–80% confluency before they were split, and were only used up to a maximum passage of 30. Cells were washed with 1x phosphate buffered saline (PBS) and detached from the flask surface with 1x PBS and 2 mM EDTA. Cells were spun down at 350 g × 4 min and the supernatant was removed prior to resuspension and counting. Cells were re-plated as needed in fresh DMEM and FBS and in the case of HEK BLUE™ hTLR4 cells, HEK Blue selection antibiotics (InvivoGen) were included every alternative passage.

Cells were transfected using the Fugene HD (Promega, Australia) transfection reagent following the manufacturer’s protocol. The Fugene database program was used to calculate the concentration of DNA and Fugene HD depending on cell type and plate used. HEK BLUE™ hTLR4 cells were also co-transfected using IRAK clones in pcDNA-DEST40 vector containing neomycin resistance gene and pcDNA 6.2/nGFP-DEST control vector at 1:10 ratio to observe transfection efficiency. Transfection efficiency was observed by visualising the green fluorescence of the reporter GFP attached to the C-terminal of the gene of interest and images were taken with a CoolSNAPFX camera (Photometrics, Arizona) attached to an Eclipse TE-2000E microscope (Nikon, Japan). Adherent cells were grown in a 6 well plate to a confluency of about 70–80% and lifted and spun down. Cell pellets were resuspended in lysis buffer (150 mM NaCl, 1% (v/v) Triton X-100, 0.1% (w/v) SDS, 50 mM Tris base at pH 8 and protease inhibitor) and separated by SDS-PAGE and detected as described above. FACS analysis was performed with a FACS Canto II analyser (BD Science, Australia). Before FACS analysis, cell suspensions were cleared of clumps by passing through a 70 μm strainer (BD Biosciences, Australia). Number of live GFP positive cells were compared to the number of non-transfected cells and the percentage of transfected live cells noted.

### Detection of cGMP

Cyclic GMP was determined in *in vitro* reactions performed with recombinant proteins and also in cell samples. In recombinant protein assays, cGMP was generated by incubating 5 µg–10 µg recombinant protein in 50 mM Tris-HCl pH 8.0, 5 mM MgCl_2_ or 5 mM MnCl_2_, 2 mM of the phosphodiesterase inhibitor isobutyl methyl xanthine (IBMX) and 1 mM GTP in a final reaction volume of 100 µl. As a negative control reaction mixture was prepared with no protein added. Reactions were incubated for 15 min at room temperature (~25 °C) and terminated with 10 mM EDTA. Tubes were boiled for 3 min, cooled on ice for 2 min and centrifuged at 4300 × g for 3 min at 4 °C^44^. The clarified supernatant was retained and assayed for cGMP content as described using ELISA based assays or mass spectrometry. The Amersham cGMP Enzyme immunoassay Biotrak (EIA) System (GE Healthcare, UK) was used to detect cGMP in the protein preparation and cell lysates, following the protocol which measures total cellular cGMP using novel lysis reagents, described by the supplier. Absorbance was measured as optical density readings at 405 or 450 nm. All cGMP quantification assays were carried out as independent triplicate experiments. Alternatively, cGMP samples were prepared as outlined above and samples were freeze-dried using the Dynavac freeze dryer and sent to the King Abdullah University of Science and Technology (KAUST) for liquid chromatography–tandem mass spectrometry (LC - MS/MS). In cell studies, HEK 293 T cells were grown for 48 hours post transfection in a Corning® Costar® cell culture 6 well plate (Sigma Aldrich, Australia), cells were lysed by the addition of 0.5% dodecyl trimethylammonium bromide supplied in the Amersham cGMP Enzyme immunoassay Biotrak (EIA) System kit. Cells were agitated on a microplate shaker for 10 minutes to facilitate lysis. Lysed cells were used immediately in the immunoassay.

### SEAP assay

HEK BLUE™ hTLR4 cells (Invivogen) were split at approximately 1.4 × 10^5^ cells per ml in a Corning® Costar® cell culture clear flat bottom 96 well plate and induced with 10 ng/μl of lipoplysaccharide (*Escherichia coli* 055:B5, Sigma-Aldrich). Control HEK BLUE™ hTLR4 cells were untreated. Cells were grown for 20–24 hours at 37 °C in DMEM medium supplemented with FBS as described above in a 5% CO_2_ incubator. 20 μl of supernatant from inducted cells was transferred in triplicate to a clear flat bottom 96 well plate and 180 μl of QUANTI Blue was added as described in the HEK BLUE™ hTLR4 QUANTI Blue^TM^, TLR4 stimulation protocol. The plate was then incubated at 37 °C for 1 hour. SEAP levels were determined spectrophotometrically at 660 nm using the Envision 2101 plate reader (Perkin Elmer, USA).

### Quantitative PCR

Transfected and non-transfected HEK BLUE™ hTLR4 cells were split at ~2 × 10^5^ cells per ml in a flat bottom 6 well plate and left as untreated controls or induced with 10 ng/μl lipopolysaccharide (*Escherichia coli* 055:B5, Sigma-Aldrich, USA) and grown further 24 h at 37 °C in 5% CO_2_. Cells were detached from the surface with 1X PBS and 2 mM EDTA, spun down at 350 g × 4 min and cell pellets were collected and stored at −80 °C. RNA was extracted from the cells using the QIAGEN RNeasy mini kit (QIAGEN, Germany) following the manufacturer’s protocol. Each sample received TURBO DNAse treatment (Ambion, Life Technologies, Germany). The amount of RNA extracted was quantified using the Nanodrop® ND-1000 Spectrophotometer, then stored at −80 °C till use. First-Strand cDNA was synthesised from 1 μg of total RNA using the Superscript™ III Reverse transcriptase (Invitrogen, Australia) using supplied oligo-dTs and dNTPs with cDNA was stored at −80 °C till use. IRAK primer pairs (Supplementary Table 1) were designed using the IDT (Integrated DNA Technologies, USA) qPCR primer design website (http://www.idtdna.com/Primerquest/Home/Index), and the specific exons of each sequence were obtained from the NCBI website. The primers were chosen from the recommended list of primers based on the expected size of the product (100–300 bp). All primer pairs were checked with Primer-BLAST (https://www.ncbi.nlm.nih.gov/tools/primer-blast/index.cgi?LINK_LOC=BlastHome) for specificity to the PCR templates. The mastermix was prepared using the SensiMix™ SYBR® Hi-ROX Kit (Bioline, Australia). Non-template controls were prepared for each qPCR analysis, consisting of RNA samples where reverse transcriptase was not added to ensure that DNA contamination of samples was not affecting qPCR results. Each sample was analysed in triplicate. The efficiency of reactions was determined using linear regression of the Log (fluorescence) per cycle number data with the LinRegPCR program version 2015.2^67,68^ which considers the amplification with the efficiency of the primer pairs. Baseline correction was performed by subtracting the fluorescence value of the mean for the first five cycles to all values. Expression data of samples was calculated relative to the two housekeeping genes (β-actin and GAPDH) expressed as previously described^69^ using the following ratio, where Ct is the crossing point threshold of the sample for the amplified genes:

$${\rm{Ratio}}={({{\rm{Efficiency}}}_{{\rm{reference}}})}^{{\rm{Ct}},{\rm{reference}}}/{({{\rm{Efficiency}}}_{{\rm{sample}}})}^{{\rm{Ct}},{\rm{sample}}}$$

### IRAK3 homology model and docking studies

Alignment of the IRAK3 amino acid sequence in different species was undertaken using the Clustal W2 multiple sequence alignment tool (https://www.ebi.ac.uk/). The IRAK3 sequences used in the alignment were obtained from the NCBI database. An IRAK3 database search for SNP variants was performed using the ExAC Browser (Beta), Exome Aggregation Consortium^70^. The crystal structure of the IRAK4 kinase domain PDB code: 2NRU^45^ was used in the alignment and homology model design of the IRAK3 pseudokinase domain as previously described^11^. Once the mutation was incorporated into the structure, a minimisation of all the hydrogens was undertaken, followed by minimisation of the side chain of either the mutation and where necessary the side chains of the surrounding residues. Molecular docking was performed using the Glide module within the Maestro molecular modelling package (Maestro version 9.3, Schrödinger, LLC, New York, USA). GTP was docked using the XP algorithm into the IRAK3 homology model. To provide an initial position for GTP in the binding site, the location of GTP within the structure of a related protein, cAMP-dependent protein kinase (PDB code: 1ATP) was used as a guide. Default parameters were applied for each docking experiment and the same methods were used for the G361L and R372L mutants of IRAK3.

### Statistical analysis

Data was analysed using GraphPad prism 7 software (GraphPad Software, USA) by t-test with Welch’s correction or one-way ANOVA followed by multiple comparison post hoc tests where P < 0.05 was considered to be significant.

## Supplementary information

Supplemenatary material

## Data Availability

The datasets generated and/or analysed during the current study are available from the corresponding author on reasonable request.

## References

[1] Halls ML, Cooper DMF (2017). Adenylyl cyclase signalling complexes – Pharmacological challenges and opportunities. Pharmacol Therapeut..

[2] Leroy J, Vandecasteele G, Fischmeister R (2018). Cyclic AMP signaling in cardiac myocytes. Curr. Opin. Physiol..

[3] Musheshe N, Schmidt M, Zaccolo M (2018). cAMP: From long-range second messenger to nanodomain signalling. Trends Pharmacol. Sci..

[4] Subramanian, H. *et al*. Distinct submembrane localisation compartmentalises cardiac NPR1 and NPR2 signalling to cGMP. *Nature Commun*. **9**, 10.1038/s41467-018-04891-5 (2018). 10.1038/s41467-018-04891-5 PMC6014982 29934640

[5] Gehring, C. & Turek, I. S. Cyclic nucleotide monophosphates and their cyclases in plant signaling. *Front*. *Plant Sci*. **8**, 10.3389/fpls.2017.01704 (2017). 10.3389/fpls.2017.01704 PMC5632652 29046682

[6] Irving, H. R., Cahill, D. M. & Gehring, C. Moonlighting proteins and their role in the control of signaling microenvironments, as exemplified by cGMP and phytosulfokine receptor 1 (PSKR1). *Front*. *Plant Sci*. **9**, 10.3389/fpls.2018.00415 (2018). 10.3389/fpls.2018.00415 PMC5883070 29643865

[7] Kwezi L (2007). The *Arabidopsis thaliana* brassinosteroid receptor (AtBRI1) contains a domain that functions as a guanylyl cyclase *in vitro*. PLoS One.

[8] Kwezi L (2011). The phytosulfokine (PSK) receptor is capable of guanylate cyclase activity and enabling cyclic GMP-dependant signaling in plants. J. Biol. Chem..

[9] Muleya V (2016). Phosphorylation of the dimeric cytoplasmic domain of the phytosulfokine receptor, PSKR1. Biochem. J..

[10] Wheeler JI (2017). The brassinosteroid receptor BRI1 can generate cGMP enabling cGMP-dependent downstream signaling. Plant J..

[11] Freihat L, Muleya V, Manallack DT, Wheeler JI, Irving HR (2014). Comparison of moonlighting guanylate cyclases – roles in signal direction?. Biochem. Soc. Trans..

[12] Jain A, Kaczanowska S, Davila E (2014). IL-1 receptor-associated kinase signaling and its role in inflammation, cancer progression, and therapy resistance. Front. Immunol..

[13] Gay NJ, Symmons MF, Gangloff M, Bryant CE (2014). Assembly and localization of Toll-like receptor signalling complexes. Nature Rev. Immunol..

[14] Lin S-C, lo Y-C, Wu H (2010). Helical assembly in the MyD88-IRAK4-IRAK2 complex in TLR/IL-1R signalling. Nature.

[15] Kobayashi K (2002). IRAK-M is a negative regulator of toll-like receptor signaling. Cell.

[16] Dossang ACG (2016). The N-terminal loop of IRAK-4 death domain regulates ordered assembly of the Myddosome signalling scaffold. Sci. Rep..

[17] Du J (2014). The structure function of the death domain of human IRAK-M. Cell Commun. Sig.

[18] Zhou H (2013). IRAK-M mediates Toll-like receptor/IL-1R-induced NFkappaB activation and cytokine production. EMBO J..

[19] Zhou H (2016). IRAKM-Mincle axis links cell death to inflammation: pathoophysiological implications for chronic alcoholic liver disease. Hepatology.

[20] Miyata M (2015). Glucocorticoids suppress inflammation via the upregulation of negative regulator IRAK-M. Nature Commun..

[21] Xiong H (2004). Inhibition of interleukin-12 p40 transcription and NF-kB activation by nitric oxide in murine macrophages and dendritic cells. J. Biol. Chem..

[22] Lucas KA (2000). Guanylyl cyclases and signaling by cyclic GMP. Pharmacol. Rev..

[23] Ahluwalia A (2004). Antiinflammatory activity of soluble guanylate cyclase: cGMP-dependent down-regulation of P-selectin expression and leukocyte recruitment. Proc. Natl. Acad. Sci. USA.

[24] Nunes AKS (2015). Involvement of AMPK, IKba-NFkB and eNOS in the sildenafil and anti-inflammatory mechanism in a demyelination model. Brain Res..

[25] Raposo C, Luna RL, Nunes AK, Thome R, Peixoto CA (2014). Role of iNOS-NO-cGMP signaling in modulation of inflammatory and myelination processes. Brain Res. Bull..

[26] Rizzo NO (2010). Reduced NO-cGMP signaling contributes to vascular inflammation and insulin resistance induced by high-fat feeding. Arterioscler. Thromb. Vasc. Biol..

[27] Sanyal A (2017). Interplay between obesity-induced inflammation and cGMP signaling in white adipose tissue. Cell Rep..

[28] Schmidtko A (2008). cGMP produced by NO-sensitive guanylyl cyclase essentially contributes to inflammatory and neuropathic pain by using targets different from cGMP-dependent protein kinase I. J. Neurosci..

[29] Tateya S (2011). Endothelial NO/cGMP/VASP signaling attenuates Kupffer cell activation and hepatic insulin resistance induced by high-fat feeding. Diabetes.

[30] Siednienko J, Nowak J, Moynagh PN, Gorczyca WA (2011). Nitric oxide affects IL-6 expression in human peripheral blood mononuclear cells involving cGMP-dependent modulation of NF-κB activity. Cytokine.

[31] Maa M-C (2008). Requirment of inducible nitric-oxide synthase in lipolysaccharide-mediated Src induction and macrophage migration. J. Biol. Chem..

[32] Harbrecht BG, Wang SC, Simmons RL, Billiar TR (1995). Cyclic GMP and guanylate cyclase mediate lipopolysaccharide-induced Kupffer cell tumor necrosis factor-alpha synthesis. J. Leukocyte Biol..

[33] Ernst PB, Carvunis A-R (2018). Of mice, men and immunity: a case for evolutionary systems biology. Nature Immunol..

[34] Buys ES (2018). Discovery and development of next generation sGC stimulators with diverse multidimensional pharmacology and broad therapeutic potential. Nitric Oxide Biol. Chem..

[35] Hollas MA, Ben Aissa M, Lee SH, Gordon-Blake JM, Thatcher GRJ (2019). Pharmacological manipulation of cGMP and NO/cGMP in CNS drug discovery. Nitric Oxide Biol. Chem..

[36] Kobayashi Y (2010). The regulatory role of nitric oxide in proinflammatory cytokine expression during the induction and resolution of inflammation. J. Leukocyte Biol..

[37] Park, M., Sandner, P. & Krieg, T. cGMP at the centre of attention: emerging strategies for activating the cardioprotective PKG pathway. *Basic Res*. *Cardiol*. **113**, 10.1007/s00395-018-0679-9 (2018). 10.1007/s00395-018-0679-9 PMC5954070 29766323

[38] Rappaport, J. A. & Waldman, S. A. The guanylate cyclase C-cGMP signaling axis opposes intestinal epithelial injury and neoplasia. *Front*. *Oncol*. **8**, 10.3389/fonc.2018.00299 (2018). 10.3389/fonc.2018.00299 PMC6091576 30131940

[39] Qi Z (2010). Ca^2+^ signaling by plant *Arabidopsis thaliana* Pep peptides depends on AtPepR1, a receptor with guanylyl cyclase activity, and cGMP-activated Ca^2+^ channels. Proc. Natl. Acad. Sci. USA.

[40] Gross I, Durner J (2016). In search of enzymes with a role in 3′,5′-cyclic guanosine monophosphate metabolism in plants. Front. Plant Sci..

[41] Marondedze, C., Wong, A., Thomas, L., Irving, H. & Gehring, C. In: Non-canonical Cyclic Nucleotides, *Handbook of Experimenal Pharmacology*. Seifert, R. ed. Cham: Springer International Publishing, pp. 87–103 (2017).

[42] Spangler CM (2009). Sensitive assay for the detection of cyclic nucleotides by mass spectrometry. BMC Pharmacol..

[43] Wesche H (1999). IRAK-M is a novel member of the Pelle/interleukin-1 receptor-associated kinase (IRAK) family. J. Biol. Chem..

[44] Muleya V (2014). Calcium is the switch in the moonlighting dual function of the ligand-activated receptor kinase phytosulfokine receptor 1. Cell Commun. Sig.

[45] Wang Z (2006). Crystal structures of IRAK-4 kinase in complex with inhibitors: a serine/threonine kinase with tyrosine as a gatekeeper. Structure.

[46] Wong A, Gehring C, Irving HR (2015). Conserved functional motifs and homology modeling to predict hidden moonlighting functional sites. Front. Bioengin. Biotech.

[47] Wong A, Gehring C (2013). The *Arabidopsis thaliana* proteome harbors undiscovered multi-domain molecules with functional guanylyl cyclase catalytic centers. Cell Commun. Sig.

[48] del Fresno C (2004). Nitric oxide activates the expression of IRAK-M via the release of TNF-a in human monocytes. Nitric Oxide.

[49] González-León MC (2006). Nitric oxide induces SOCS-1 expression in human monocytes in a TNF-α-dependent manner. J. Endotoxin Res..

[50] Peng HB, Libby P, Liao JK (1995). Induction and stabilization of I kappa B alpha by nitric oxide mediates inhibition of NF-kappa B. J. Biol. Chem..

[51] Kim M-Y (2008). Downregulation by lipopolysaccharide of Notch signaling, via nitric oxide. Journal of Cell Science.

[52] Ludidi NN, Gehring C (2003). Identification of a novel protein with guanylyl cyclase activity in *Arabidopsis thaliana*. J. Biol. Chem..

[53] Xu N, Fu D, Li S, Wang Y, Wong A (2018). GCPred: a web tool for guanylyl cyclase functional center prediction from amino acid sequence. Bioinformatics.

[54] Muleya V, Irving HR (2016). Delineating a new class of membrane-bound guanylate cyclases. Springer Sci. Rev..

[55] Ladwig F (2015). Phytosulfokine regulates growth in Arabidopsis through a response module at the plasma membrane that includes Cyclic Nucleotide-Gated Channel17, H-ATPase, and BAK1. Plant Cell.

[56] Carlton VE, Ireland JS, Useche F, Faham M (2006). Functional single nucleotide polymorphism-based association studies. Human Genomics.

[57] Hardison RC (2003). Comparative Genomics. PLoS Biol..

[58] Liu Y, Ruoho AE, Rao VD, Hurley JH (1997). Catalytic mechanism of the adenylyl and guanylyl cyclases: modeling and mutational analysis. Proc. Natl. Acad. Sci. USA.

[59] McCue LA, McDonough KA, Lawrence CE (2000). Functional classification of cNMP-binding proteins and nucleotide cyclases with implications for novel regulatory pathways in *Mycobacterium tuberculosis*. Genome Res..

[60] Kawai T, Akira S (2007). Signaling to NF-κB by Toll-like receptors. Trends Mol. Med..

[61] Yin Q, Fu T-M, Li J, Wu H (2015). Structural biology of innate immunity. Ann. Rev. Immunol.

[62] Johannessen CM (2010). COT drives resistance to RAF inhibition through MAP kinase pathway reactivation. Nature.

[63] Muleya V, Wheeler JI, Irving HR (2013). Structural and functional characterization of receptor kinases with nucleotide cyclase activity. Meth. Mol. Biol..

[64] Lee, N. Molecular aspects of ara regulation. In *The Operon* (eds Miller, J. H. & Rezinkoff, W. S.) 389–410 (Cold Spring Harbor Laboratory, 1980).

[65] Lee N, Francklyn C, Hamilton EP (1987). Arabinose-induced binding of AraC protein to *ara*I2 activates the *ara*BAD operon promoter. Proc. Natl. Acad. Sci. USA.

[66] Schleif FW (1992). DNA looping. Ann. Rev. Biochem.

[67] Ruijter JM, LorenzJari P, Tuomi JM, Hecker M, van den Hoff MJB (2014). Fluorescent-increase kinetics of different fluorescent reporters used for qPCR depend on monitoring chemistry, targeted sequence, type of DNA input and PCR efficiency. Microchimica Acta.

[68] Ruijter JM (2013). Evaluation of qPCR curve analysis methods for reliable biomarker discovery: bias, resolution, precision, and implications. Methods.

[69] Yaakob NS, Chinkwo KA, Chetty N, Coupar IM, Irving HR (2015). Distribution of 5-HT_3_, 5-HT_4_ and 5-HT_7_ receptors along the human colon. J. Neurogastroent. Mot.

[70] Lek M (2016). Analysis of protein-coding genetic variation in 60,706 humans. Nature.

