# Supplementary material for: IRAK3 modulates downstream innate immune signalling through its guanylate cyclase activity
Source: Sci Rep. 2019 Oct 29;9:15468. doi: 10.1038/s41598-019-51913-3 (PMC6820782; doi:10.1038/s41598-019-51913-3)
Supplement: Supplementary file 1 — Supplemenatary material [file 41598_2019_51913_MOESM1_ESM.pdf]

Supplementary material for

IRAK3 modulates downstream innate immune signalling through its guanylate cyclase activity

Freihat LA, Wheeler JI, Wong A, Turek I, Manallack DT and Irving HR

The following pages include:

Supplementary Table 1

Supplementary Figures 1 to 5

Supplementary Table 1. Primers used in cloning, sequencing, mutagenesis and qPCR

The primer sequences used in Gateway cloning are coloured as follows: the specific gateway sequence for recombination with entry clone is in purple, the Kozak sequence is green, the Myc tag is blue, the gene specific sequence is in black and the stop codon is in red. The primers used to sequence the genes of interest are listed under sequencing primers. The primers used in mutagenesis are listed showing the gene specific forward primer and the antisense primer each with the associated mutation underlined in red. The primers used in qPCR reactions are listed under quantitiative PCR.

Primer name	Primer sequence (5' to 3')
Gateway IRAK3-1-Fwd	ggg gac aag ttt gta caa aaa agc agg ctt cac cat ggg gat ggc ggg gaa ctg tgg ggc c
Gateway IRAK3-2- Rev	ggg gac cac ttt gta caa gaa agc tgg gtc cta ttc ttt ttt gta ctg ttc ata
Gateway IRAK3-2NS- Rev	ggg gac cac ttt gta caa gaa agc tgg gtc ttc ttt ttt gta ctg ttc ata
**Sequencing primers**	
201-Fwd	tcg cgt taa cgc tag cat gga tct c
201-Rev	gta aca tca gag att ttg aga cac
TK polyA Rev	ctt ccg tgt ttc agt tag c
T7 Fwd	taa tac gac tca cta tag gg
GFP-Rev	tgg tgc aga tga act tca gg
**Mutagenesis primers**	
IRAK3G361LFwd	tcc att aaa aca gat gtc tac agc ttt tta att gta ata atg gaa gtt cta aca gg
IRAK3G361L_antisense	cct gtt aga act tcc att att aca att aaa aag ctg tag aca tct gtt tta atg ga
IRAK3R372LFwd	gga att gta ata atg gaa gtt cta aca gga tgt tta gta gtg tta gat gat c
IRAK3R372L_antisense	gat cat cta aca cta cta aac atc ctg tta gaa ctt cca tta tta caa ttc c
**Quantitative PCR**	
IRAK3 Fwd 305	gaa caa gag aat tac ttt ggt cct g
IRAK3 Rev 414	gga ctc aac act gct cca tag
IRAK3 Fwd 974	gtg gca gta tat caa gtg caa ac
IRAK3 Rev 1519	ctc cag gaa tag agg aga agg a
IRAK4 Fwd 428	ttg ctc cca gat gct gtt c
IRAK4 Rev 532	aga ttc tgc aca ggt gtc atc
IRAK4 Fwd 885	caa gtg atg gag atg acc tct g
IRAK4 Rev 1492	agc agc tgt tga acc ttc tta
GAPDH Fwd	gaa ggt gaa ggt cgg agt c
GAPDH Rev	gaa gat ggt gat ggg att tc
β-actin Fwd	gcc ctg agg cac tct tcc a
β-actin Rev	ttg cgg atg tcc acg tca

A Search motif: [RKS][YFW][GCTH][VIL][FV]X{3}[VIL]X{4}[KR]X{2,3}[DE]

```
IRAK1:   LPEEYIKTGRLAVDTDTFSFGVVVLETLAGQRAVKTHGARTKYLKDLVE
IRAK2:   LPEDFIRVGQLTKRVDIFSCGIVLAEVLTGIPAMDNN-RSPVYLKDLLL
IRAK3:   MPEEYIRQGKLSIKTDVYSFGIVIMEVLTGCRVVLDD-PKHIQLRDLLR
IRAK4:   MAPEALR-GEITPKSDIYSFGVVLLEIITGLPAVDEH-REPQLLLDIKE
                           12345---9----14—17/18
```

B

```
H. sapiens:      344 EYIRQGKLSIKTDVYSFGIVIMEVLTGCRVVLDDPKHIQLRDLLRELMEK 393
P. troglodytes:  344 EYIRQGKLSIKTDVYSFGIVIMEVLTGCRVVLDDPKHIQLRDLLRELMEK 393
M. mulata:       344 EYIRQGKLSIKTDVYSFGIVIMEVLTGCRVVLDDPKHIQLRDLLIELMEK 393
C. lupus:        537 EYIRQGKLSIKTDVYSFGIVIMEVLTGCKVVLDDPKHIQLRDLLMELMEK 586
B. taurus:       252 EYIRQGKLSIKTDIYSFGIVIMEVLTGCKVVLDEPKHIQLRDLLIELMEK 301
M. musculus:     357 EYIRQGRLSVKTDVYSFGIVIMEVLTGCKVVLDDPKHVQLRDLLMELMEK 406
R. novegicus:    358 EYIRQGRLTIKTDVYSFGIVIMEVLTGCKVVLDDPKHVQLRDLLMELVEK 407
D. rerio:        350 EYIRDGKLSVKLDVYSLGVVILETCTGQKVKQESSKNLFL---------- 389
                                    1-3----------14
```

Supplementary Figure 1. Alignment of guanylate cyclase centre in IRAK3.

A. Alignment of human IRAK family members (IRAK1: AAH54000.1, IRAK2: NP_001561.3, IRAK3: NP_009130.2 and IRAK4: NP_001338274.1) showing the amino acids present in the guanylate cyclase centre. The original search motif is shown for reference. The amino acids in purple are predicted to be important in guanylate cyclase activity while those shown in green are predicted to be important in binding the essential metal ion cofactors Mg^{2+} or Mn^{2+}. The amino acids in underlined red are different to the guanylate cyclase search motif and show why IRAK1, IRAK2 and IRAK4 were not detected by the original search. Multiple Sequence Alignment of the amino acid sequences obtained from NCBI was done using Clustal Omega [1].

B. Alignment of IRAK3 amino acid residues from different organisms showing the guanylate cyclase centre outlined in purple with the region highlighted in yellow being important for guanylate cyclase function. The sequences are from *Homo sapiens* (NP_009130.2), *Pan troglodytes* (XP_522458.2), *Macaca mulatta* (XP_001117080.1), *Canis lupus* (XP_005625714.1), *Bos taurus* (NP_001177228.1), *Mus musculus* (NP_082955.2), *Rattus norvegicus* (NP_001101571.1) and *Danio rerio* (XP_003198307.2). The alignment was performed on the homologene site at NCBI using MUSCLE [2].

1 Chojnacki, S., Cowley, A., Lee, J., Foix, A. & Lopez, R. Programmatic access to bioinformatics tools from EMBL-EBI update: 2017. *Nucl. Acid Res.* **45**, W550-W553 (2017).

2 Edgar, R. C. MUSCLE: multiple sequence alignment with high accuracy and high throughput. *Nucl. Acid Res.* **32**, 1792-1797 (2004).

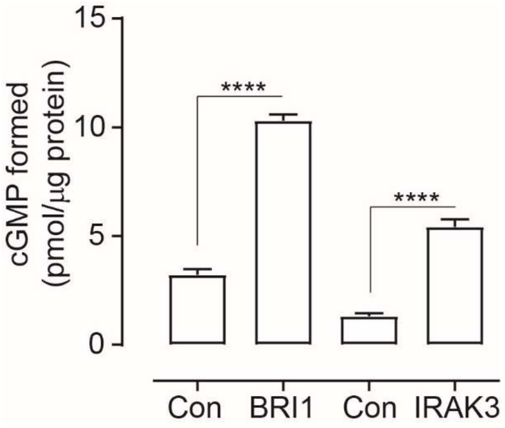

Supplementary Figure 2. Mass spectrometry analysis of cGMP generated by IRAK3.
Liquid chromatography tandem mass spectrometry (LC-MS/MS) analysis of cGMP produced by protein preparations of recombinant IRAK3 in the presence of Mn^{2+} (preferred cofactor see Figure 1) and recombinant BRI1 in the presence of Mg^{2+} (preferred cofactor (Kwezi et al., 2007; Wheeler et al., 2017). The content of cGMP generated by each of the proteins in 3 separate reactions was quantified. Compared to the corresponding 'no protein' controls (Con) both IRAK3 and BRI1 produce significant amounts of cGMP (mean ± sem of three technical replicates, **** $P < 0.0001$ t-test with Welch's correction). These results were confirmed in separate reactions with a different protein preparation of BRI1 and IRAK3.

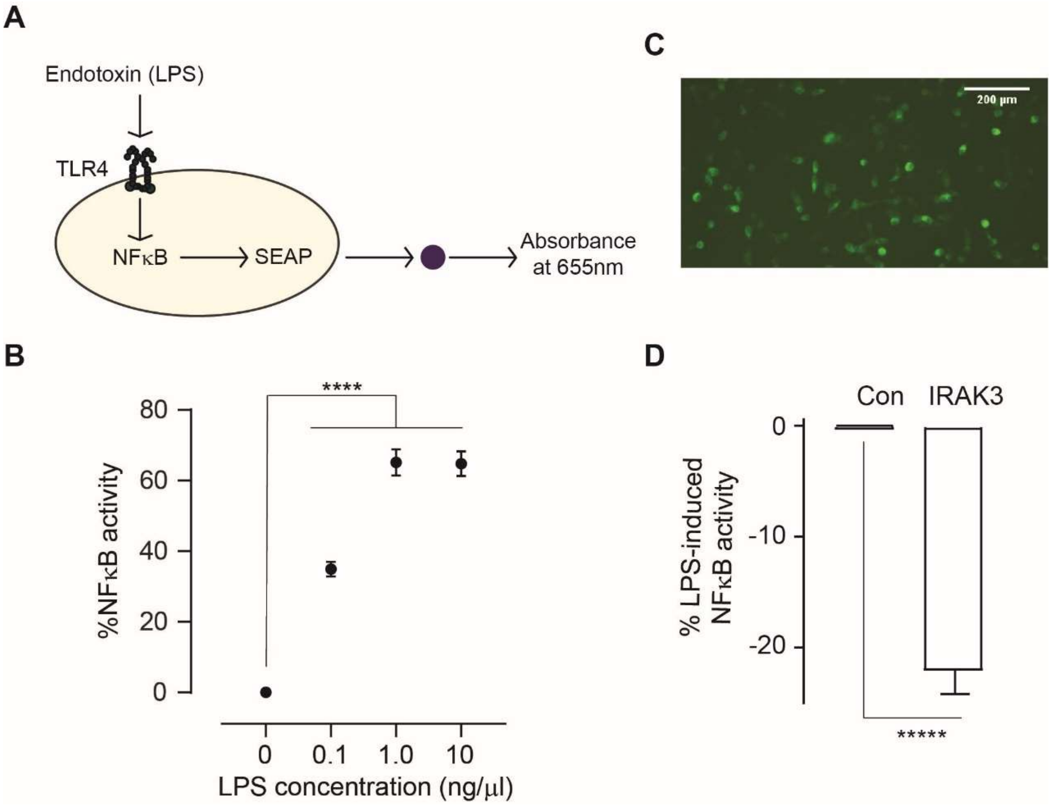

Supplemenatary Figure 3. SEAP assay in transfected HEK BLUE hTLR4 cells.

A. The basis of the SEAP assay reporting activation of NFκB in response to an endotoxin like lipopolysaccharide (LPS). HEK BLUE hTLR4 cells contain the human toll-like receptor 4 (TLR4), lymphocyte antigen 96 (MD-2) and the cluster of differentiation (CD14) co-receptor genes, and an inducible secreted embryonic alkaline phosphatase (SEAP) reporter gene. The SEAP reporter gene is under the control of the IL-12 p40 promoter fused to five NFκB (nuclear factor kappa-light-chain-enhancer of activated B cells) and AP-1 (activator protein 1) binding sites. Diagram adapted from http://www.invivogen.com/hek-blue-lps-detection-kit (last accessed 8/07/2015).

B. NFκB activity in HEK BLUE hTLR4 cells after 24 h treatment with LPS. HEK BLUE hTLR4 cells were induced with different concentrations of LPS and resultant NFκB activity compared to the non-induced cells (mean ± sem, n = 8, **** $P < 0.0001$ one-way ANOVA followed by Dunnett's multiple comparison test).

C. HEK BLUE hTLR4 cells transfected with plasmid expressing C-terminally GFP-tagged wildtype (WT) IRAK3. Scale bar = 200 µm.

D. Effect of IRAK3 transfection on the LPS-induced NFκB activity of HEK BLUE hTLR4 cells. HEK BLUE hTLR4 cells were transfected with plasmid expressing C-terminally GFP-tagged wildtype (WT) IRAK3. Cells were induced with lipopolysaccharide (LPS, 10 ng/µl) and after 24 hours SEAP activity correlating to NFκB induction was measured and the activity was normalised to the control vector containing GFP only (Con). Cells expressing IRAK3 suppressed LPS-induced NFκB activity relative to the control cells (Con) set at zero (mean ± sem, n = 12 **** $P < 0.0001$, t-test with Welch's correction).

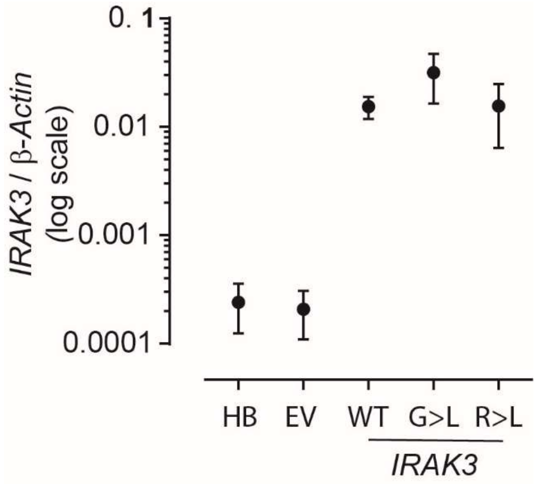

Supplementary Figure 4. Quantification of *IRAK3* gene expression in transected HEK BLUE hTLR4 cells. Reverse transcriptase quantitative PCR was used to validate the expression of *IRAK3* RNA in HEK BLUE hTLR4 cells only (HB) and following transfection with empty vector (EV), wildtype *IRAK3* (WT) or *IRAK3* mutant G361L (G>L) or *IRAK3* mutant R372L (R>L) constructs. Data was expressed relative to *β-actin* (mean ± sem, n = 2-3).

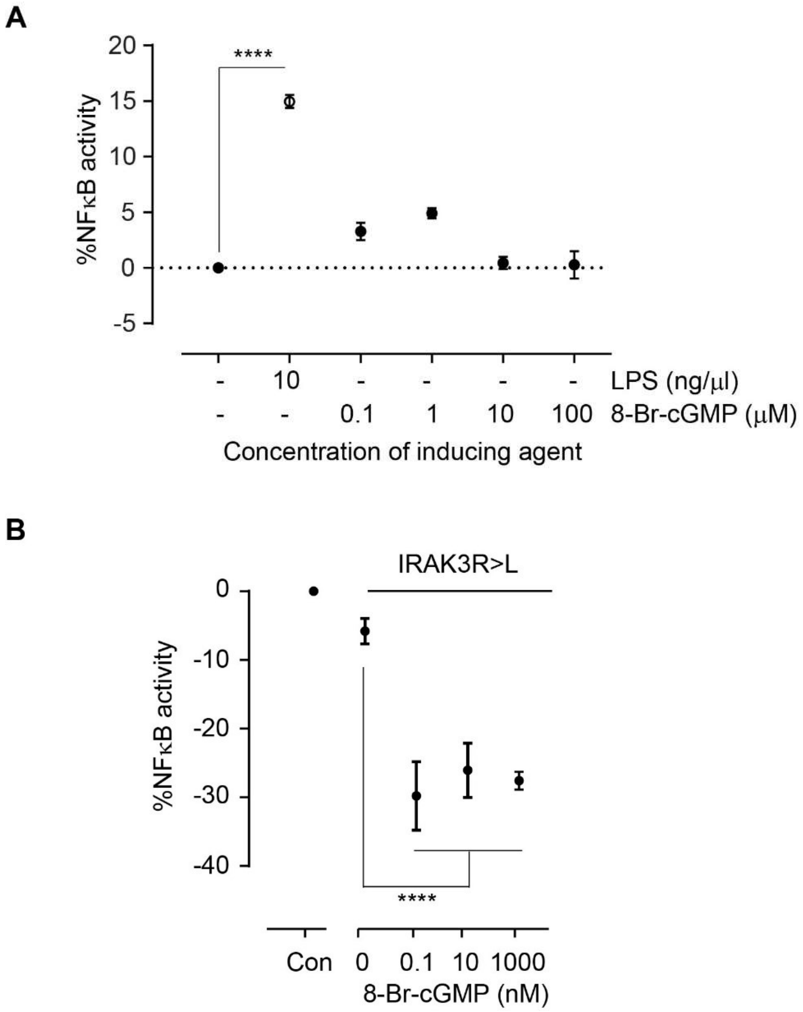

Supplemenatary Figure 5. Effects of 8-bromo-cGMP on NFκB activity in HEK BLUE hTLR4 cells.

A. Untransfected HEK BLUE hTLR4 cells were treated with increasing concentrations of membrane permeable 8-bromo-cGMP (8-Br-cGMP) in the absence or presence of lipopolysaccharide (LPS). All values of NFκB activity were compared to the uninduced control set at zero percent (mean ± sem, n = 6 - 8, **** P < 0.0001, one-way ANOVA followed by Dunnett's multiple comparison test).

B. Effects of membrane permeable 8-Br-cGMP on LPS induced NFκB activity in HEK BLUE hTLR4 cells transfected with IRAK3 mutant R372L (IRAK3R>L). The treatments are compared to LPS response of untransfected HEK BLUE hTLR4 cells (Con) set at zero percent. (mean ± sem, n = 4 - 6, **** P < 0.0001, one-way ANOVA followed by Dunnett's multiple comparison test).
